# Identification of the Bcl-2 and Bax homologs from *Rhipicephalus haemaphysaloides* and their function in the degeneration of tick salivary glands

**DOI:** 10.1186/s13071-021-04879-z

**Published:** 2021-08-04

**Authors:** Shanming Hu, Yanan Wang, Zhengmao Xu, Yongzhi Zhou, Jie Cao, Houshuang Zhang, Jinlin Zhou

**Affiliations:** Key Laboratory of Animal Parasitology of Ministry of Agriculture, Shanghai Veterinary Research Institute, Chinese Academy of Agricultural Sciences, Shanghai, 200241 China

**Keywords:** Salivary gland degeneration, Apoptosis, Bcl-2, Bax

## Abstract

**Background:**

The salivary glands of female ticks degenerate rapidly by apoptosis and autophagy after feeding. Bcl-2 family proteins play an important role in the apoptosis pathways, but the functions of these proteins in ticks are unclear. We studied Bcl-2 and Bax homologs from *Rhipicephalus haemaphysaloides* and determined their functions in the degeneration of the salivary glands.

**Methods:**

Two molecules containing conserved BH (Bcl-2 family homology) domains were identified and named *RhBcl-2* and *RhBax*. After protein purification and mouse immunization, specific polyclonal antibodies (PcAb) were created in response to the recombinant proteins. Reverse transcription quantitative PCR (RT-qPCR) and western blot were used to detect the presence of *RhBcl-2* and *RhBax* in ticks. TUNEL assays were used to determine the level of apoptosis in the salivary glands of female ticks at different feeding times after gene silencing. Co-transfection and GST pull-down assays were used to identify interactions between RhBcl-2 and RhBax.

**Results:**

The RT-qPCR assay revealed that *RhBax* gene transcription increased significantly during feeding at all tick developmental stages (engorged larvae, nymphs, and adult females). Transcriptional levels of *RhBcl-2* and *RhBax* increased more significantly in the female salivary glands than in other tissues post engorgement. *RhBcl-2* silencing significantly inhibited tick feeding. In contrast, *RhBax* interference had no effect on tick feeding. TUNEL staining showed that apoptosis levels were significantly reduced after interference with *RhBcl-2* expression. Co-transfection and GST pull-down assays showed that *RhBcl-2* and *RhBax* could interact but not combine in the absence of the BH3 domain.

**Conclusions:**

This study identified the roles of *RhBcl-2* and *RhBax* in tick salivary gland degeneration and finds that the BH3 domain is a key factor in their interactions.

**Graphical Abstract:**

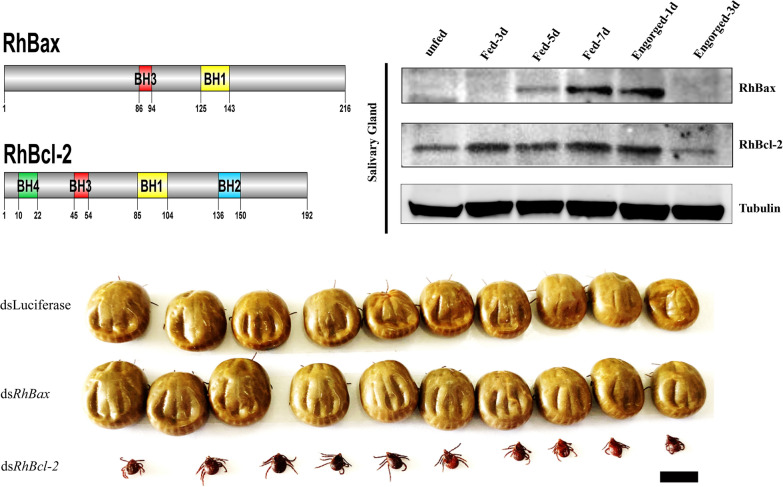

**Supplementary Information:**

The online version contains supplementary material available at 10.1186/s13071-021-04879-z.

## Background

Ticks are obligate blood-feeding arthropods and vectors of many pathogens [1]. They usually stay attached to their hosts and feed for several days or even weeks [2]. The salivary glands of ticks mediate diverse functions that ensure tick survival [3]. Tick-borne pathogens (TBP) are transmitted to the host by the saliva used during tick feeding [4].

The salivary glands of female ticks degenerate rapidly within 4 days after engorgement [5]. Salivary gland degeneration begins at the early stage of rapid feeding and may be caused by apoptosis and autophagy related to the increase of ecdysone in the hemolymph [6, 7]. During degeneration, the granular acini undergo DNA fragmentation and caspase enzyme activity increases [8, 9]. According to the comprehensive analysis of the global protein changes, some proteins associated with apoptosis and autophagy changed and some proteins linked to the degradation of DNA and proteins were consistently upregulated [10]. However, the mechanism of these effects is unknown.

Apoptosis is an important part of programmed cell death (PCD). Apoptosis is programmed to transmit death induction signals and carry out the biochemical processes of cell decomposition by a group of specific interacting proteins [11]. For embryonic development, tissue homeostasis, and organism stability in multicellular organisms, apoptosis is indispensable and is a normal physiological process [12]. During apoptosis, a decrease in cell volume, cytoplasmic condensation, mitochondrial membrane permeabilization, DNA fragmentation, and chromatin condensation followed by nuclear fragmentation and cytoplasmic membrane blebbing take place, which leads to complete cell division. In the late stage of apoptosis, apoptotic bodies are produced due to cell fragmentation [13].

Apoptosis is caused by sequential activation of cysteine proteases of the caspase family in two distinct but convergent pathways [14]. The extrinsic pathway, also known as the death receptor pathway, activates caspase-8 when the “death receptor” Fas binds to its ligand FasL, and then recruits Fas-associated death domain (FADD) proteins and caspase-8 enzymes in the cytoplasm to form a death-inducing signaling complex (DISC) [15]. The intrinsic pathway (also termed the mitochondrial pathway) can be triggered by the proapoptotic proteins of the B-cell lymphoma 2 (Bcl-2) family. These cause mitochondrial membrane permeabilization to release mitochondrial cytochrome *c* into the cytoplasm, further activating caspases to induce apoptosis [16]. These initiator caspases can cleave and activate the effector caspases (caspase-3, caspase-6, and caspase-7) that mediate cellular demolition by cleaving multiple critical cellular proteins [17].

The Bcl-2 protein family is involved in apoptosis via the intrinsic pathway. Bcl-2 was first discovered via a chromosome translocation that is the hallmark of human follicular lymphoma, and it was subsequently found to promote cell survival as an antiapoptotic protein of the Bcl-2 protein family [18]. Its homologs Bcl-X, Bcl-w, Mcl-1, and A1 were also found to inhibit apoptosis, but Bax, Bak, and Bok promoted apoptosis. The Bcl-2 homology (BH) domain is a vital functional domain for the interaction of the Bcl-2 family proteins. The BH3 domain was found to be a key domain for Bax, Bak, and Bok to promote apoptosis [19]. Bim, Bid, Bad, Puma, and Noxa only had the BH3 domain, so they were termed BH3-only proteins.

The Bcl-2 family proteins regulate apoptosis mainly by regulating mitochondrial membrane permeability, either alone or in collaboration with other proteins. For example, Bax forms oligomers on the mitochondrial membrane that perturb membrane integrity and promote apoptosis, while Bcl-2 and Bcl-xL inhibit their effects by binding to Bax to form heterodimers [20, 21]. BH3-only protein can play a direct or indirect proapoptotic role. It can directly bind and activate Bax and Bak proteins to promote apoptosis, and it can also indirectly promote Bax and Bak protein expression to cause apoptosis by inhibiting the action of the anti-proteins [22, 23].

Bcl-2 family proteins exist in both mammalian and non-mammalian species. The nematode *Caenorhabditis elegans* has two members of the Bcl-2 family, EGL-1 and CED-9, that regulate cell death [24]. Two Bcl-2 family proteins (Debcl and Buffy) have high homology with Bok found in *Drosophila* [6]. Using RNA interference, Ayllon [25] injected Bcl-2 dsRNA into *Ixodes scapularis* and showed feeding disruption. This indicated that Bcl-2 plays an important role in the intrinsic apoptosis of ticks. However, the mechanism by which Bcl-2 family proteins regulate apoptosis in ticks is unknown. In this study, we identified Bcl-2 and Bax from *Rhipicephalus haemaphysaloides* and investigated their functions in the degeneration of tick salivary glands at different feeding times.

## Methods

### Tick feeding and tissue collection

Adult *R. haemaphysaloides* were collected from water baffles in May 2001 in Wuhan, Hubei Province, China. The ticks were fed on the ears of New Zealand white rabbits (SLAC, Shanghai Institutes for Biological Science, CAS), and maintained in artificial climate incubators of the Shanghai Veterinary Research Institute [26]. Tick tissues were rapidly dissected, washed with phosphate-buffered saline (PBS, PH 7.4, with 0.14 M NaCl and 0.0027 M KCl, 0.01 M phosphate buffer; Gibco, Life Technologies, Carlsbad, CA, USA), and placed in PBS or TRIzol (Invitrogen, Carlsbad, CA, USA) reagent at −80 °C.

### RNA extraction and cDNA synthesis

RNA was derived from ticks dissected at different development stages (eggs, unfed and fed larvae, nymphs, and adults) and different tissues (salivary glands, midguts, and ovaries of female ticks), and preserved in TRIzol reagent. The HiScript^®^ III RT SuperMix for qPCR (+gDNA wiper) kit (Vazyme Biotech, Nanjing, China) was used, according to manufacturer protocol.

### Cloning, sequence analysis

*RhBcl-2* and *RhBax* primers were designed based on a comparison of the salivary gland transcriptomes of starved and engorged *R. haemaphysaloides* [27, 28]. A BLAST analysis of the translation products deduced from the open reading frames (ORFs) was performed. SignalP 4.1 (http://www.cbs.dtu.dk/services/SignalP/) [29] and ExPASy (http://web.expasy.org/compute_pi/) [30] were used for the signal peptide analysis and isoelectric point (pI) prediction. We aligned *RhBcl-2* and *RhBax* with the Bcl-2 and Bax protein sequences of other species using Genetyx v.6 (GENETYX, Tokyo, Japan). For phylogenetic analysis, the alignment of the sequences was performed using the MUSCLE algorithm [31] and inferred using the maximum likelihood method with the default settings in MEGA X software [32]. Bootstrap support values were estimated using 500 bootstrap replicates [28].

### RT-qPCR analyses

The expression levels of *RhBcl-2* and *RhBax* were examined in different development stages (eggs, unfed and fed larvae, nymphs, and adults) and different tissues (salivary glands, midguts, and ovaries of female ticks) during the feeding time. The feeding time includes the early-feeding period (fed 3 days), the fast-feeding period (fed 5 to 7 days), and the end of the feeding period (engorged 1 to 3 days) [28]. After *RhBcl-2* and *RhBax* primers (Additional file 1: Table S1) designed using Primer Premier 5, the cDNAs of the above stages and tissues were analyzed by RT-qPCRs, which were conducted using ChamQ Universal SYBR qPCR Master Mix (Vazyme) green and gene-specific (Additional file 1: Table S1) primers with a QuantStudio 5 PCR System (Applied Biosystems, Austin, TX, USA). The RT-qPCR process consisted of 95 °C for 30 s, then 40 cycles of 95 °C for 5 s and 60 °C for 30 s, followed by analysis of the melting curve. All samples were analyzed in triplicate. The data used elongation factor-1 (ELF1A, GenBank Accession number AB836665) as an internal control [31], and this was used to analyze the relative gene expression in each sample by the 2^−△Ct^ method [32, 33].

### Expression of recombinant *RhBcl-2* and *RhBax*

Specific *RhBcl-2* and *RhBax* primers (Additional file 1: Table S2) were designed in a pET-30a or PGEX-4T-1 vector. The *RhBcl-2* and *RhBax* amplified PCR products were purified and digested with *BamH*I, *EcoR*I, or *Xho*I (New England Biolabs, USA) and ligated into pET-28a or PGEX-4T-1 (Invitrogen) using the In-Fusion HD Cloning Kit (Takara Clontech, Mountain View, CA, USA) [28]. These recombinants were transformed and expressed in *Escherichia coli* BL21 (DE3) strain (TIANGEN, Beijing, China). The strains were grown at 37 °C until the OD_600_ reached 0.8. After isopropyl β-d-1-thiogalactopyranoside (IPTG) was added to 1 mM, the protein expression was induced at 25 °C for 20 h. The recombinant, containing His-tagged protein, was purified by affinity chromatography using Ni–NTA His resin (Thermo Fisher Scientific, Waltham, MA, USA). The recombinant containing the glutathione-S-transferase (GST)-tagged protein was purified by affinity chromatography using resin and gravity.

### GST pull-down of RhBcl-2 and RhBax

To confirm whether RhBcl-2 interacts with RhBax, 1 mM IPTG induced expression for 8 h of pET-30a-RhBax, PGEX-4T-RhBcl-2, and PGEX-4T-1 recombinant *E. coli* BL21. The supernatant was separated using GST agarose (Merck, Darmstadt, Germany) according to manufacturer’s instructions. Anti-GST antibody (Proteintech, Chicago, IL, USA) and goat anti-mouse secondary antibody (Proteintech) were used to confirm that GST/GST-RhBcl-2 was successfully separated, and anti-His antibody (Proteintech) was used to detect the His-RhBax protein.

### Cells and transient co-transfection assays

HEK 293T cells were maintained in Dulbecco’s modified Eagle medium (DMEM, Gibco), supplemented with 8% heat-inactivated fetal bovine serum (Biological Industries, Kibbutz Beit Haemek, Israel) and 1% penicillin (Gibco) at 37 °C [28].

The full-length ORF of *RhBcl-2* was inserted into the p3×Flag-CMV-14 vector (MiaoLing Plasmid Sharing Platform, Wuhan, China) with FLAG-tag at the N-terminal with gene-specific primers (Additional file 1: Table S2). *RhBax* was inserted into the pCMV-HA vector using the same method. The fragment of *RhBax* deleted BH3 domain (*RhBax*-ΔBH3) and BH1 domain (*RhBax*-ΔBH1) was amplified by splicing by overlap extension (SOE) of recombinant PCR with specific primers (Additional file 1: Table S3), and they were inserted into the pCMV-HA vector [34]. Transfection using Lipofectamine™ 3000 Transfection Reagent (Invitrogen) was performed according to the manufacturer’s protocol with a DNA-to-lipofectamine ratio of 1: 2 w/v. The HEK 293T cells were transformed with 2.5 μg/well of plasmid or co-transfected with the equivalent amount of two different plasmids in six-well plates [28]. After 24 h, cells were washed twice with cold PBS. They were then lysed in a modified RIPA buffer (Thermo Fisher Scientific, Waltham, MA, USA) in the presence of phenylmethylsulfonyl fluoride (PMSF) (Sangon Biotech Co., Ltd., Shanghai, China), after which they were then placed on ice for 10 min on a swirling plate to ensure uniform spreading. The samples were then centrifuged at 12,000×*g* for 10 min to pellet the cell debris.

### Antibody generation

We predicted the epitopes of RhBcl-2 and RhBax online (http://www.iedb.org/), and synthesized keyhole limpet hemocyanin (KLH) [35]-coupled polypeptides based on the epitope amino acid sequences (RhBcl-2: GLQWNTCPPLPRPSK, RhBax: STPTHEETREE). Polypeptides were dissolved in PBS and Freund’s adjuvant (complete and incomplete; Invitrogen). Equal volumes of these solutions were then emulsified together and intraperitoneally injected into 6- to 8-week-old BALC/c mice provided by the Shanghai Laboratory Animal Center (Shanghai Institutes for Biological Science, Chinese Academy of Sciences, Shanghai, China). The mixtures were injected three times at 2-week intervals. Serum was collected from the posterior orbital vein on the seventh day after the third immunization. RhBcl-2 and RhBax anti-sera were used to detect RhBcl-2 and RhBax in protein extracts.

### Western blotting

The collected ticks and tick tissues were extracted using Tris-buffered saline (TBS; 10 mM Tris-HCl, pH 7.5; 150 mM NaCl with 1 mM phenylmethanesulfonyl fluoride) in the presence of phenylmethylsulfonyl fluoride (PMSF) (Sangon Biotech Co., Ltd., Shanghai, China). These samples were then placed on ice and broken by sonication. A Bradford Protein Assay Kit (TIANGEN) was used to determine the protein concentration. Proteins were separated by 10% sodium dodecyl sulphate polyacrylamide gel electrophoresis (SDS-PAGE, 12%, Genescript, Nanjing, China) and transferred onto polyvinylidene difluoride (PVDF) membranes. After blocking in TBS containing 5% skim milk, the proteins were incubated at 4 °C overnight with primary antibodies (FLAG Tag D6W5B, Cell Signaling Technology Inc., Danvers, MA, USA; HA-Tag C29F4, Cell Signaling Technology Inc.; GST Tag, 66001-2-Ig, Proteintech, Chicago, IL, USA; His Tag, 66005-1-Ig, Proteintech). Anti RhBcl-2 and RhBax sera were used to detect RhBcl-2 and RhBax in protein extracts, and anti-tubulin (66031-1-Ig, Proteintech) primary antibody was used as a constitutive control to normalize the signal from the target protein. The membranes were rinsed five times in Tris-buffered saline with Tween-20 (TBST) and incubated with a secondary antibody IgG(H+L) conjugated with HRP (goat anti-mouse, 31430, 1:5000, Thermo Fisher Scientific; goat anti-rabbit, 31460, 1:5000, Thermo Fisher Scientific) for 1 h at room temperature. The membranes were rinsed five times with TBST. The signal was detected with an Enhanced Chemiluminescent Substrate Reagent Kit (NCM Biotech, Suzhou, China). Images were captured using the ChemiDoc Touch imaging system (Bio-Rad, Hercules, CA, USA) [28].

### RNAi of *RhBcl-2* or *RhBax*

The RNAi experiments were designed against *RhBcl-2* and *RhBax* genes. For the design of RNAi primers, *RhBcl-2* and *RhBax* sequences were screened by Primer Premier 5. Specific primers (Additional file 1: Table S4) containing the T7 polymerase promoter sequence were used for PCR amplification. The amplicons were then purified to obtain templates for double-stranded RNA synthesis using the T7 RiboMAX Express RNAi system (Promega, Madison, WI, USA). The unrelated gene Luciferase dsRNA was synthesized using the same methods described previously and used as the negative control. Unfed female ticks (*n* = 20 females per group) were microinjected with approximately 1 μg of dsRNA. Control ticks were injected with unrelated Luciferase dsRNA. After dsRNA injection, ticks were held in a humidity chamber for 1 day, after which they were allowed to feed on rabbit ears until they were fully engorged. Each group (control and *RhBcl-2* or *RhBax*) of ticks was allowed to feed on three rabbits (20 ticks on each rabbit). The biological parameters analyzed were: attachment rate at 24 h (counting the number of dead females attached to rabbit ears on the next day); number of engorged ticks (counting the number of females dropped off rabbit ears on the seventh day); death rate (counting the number of dead females on the seventh day); egg laying rate (counting how many females had the ability to lay eggs on the 15th day), and hatchability rate (counting how many egg larvae hatched on the 13th day). RT-qPCR was used to evaluate gene silencing efficiency.

Interference with the salivary glands was performed using dsRNA in vitro. The salivary glands of female ticks fed on rabbit for 5 days were dissected and placed into complete L15 medium with 1% penicillin–streptomycin [36]. Each well received 5 μg of dsRNA and was incubated at 27 °C with no CO_2_ for 48 h. The control group received unrelated Luciferase dsRNA. Each treatment group included the salivary glands from 10 ticks. After incubation, the dissected salivary glands in each group were fixed in 4% paraformaldehyde for TUNEL staining or fixed in 2.5% glutaraldehyde at 4 °C for at least 24 h. The remaining salivary glands were used for western blot and RNA.

The data were obtained from three independent experiments with three biological replicates, and were used to analyze the relative gene expression in each sample by the 2^−ΔCt^ method.

### TUNEL staining

Salivary glands were fixed in 4% paraformaldehyde and embedded in paraffin. Sections (5 μm) of salivary glands were mounted on microscope slides. Sections were then deparaffinized, washed in 100% ethanol, and rehydrated. Samples were washed with PBS. After antigen retrieval with 0.1% Triton X-100, the sections were incubated for 1 h with 1:9 TdT mixed with fluorescent-labeled dUTP at 37 °C, following the instructions of the Roche In Situ Cell Death Detection Kit, POD (Roche, Mannheim, Germany). After washing 2–3 times with PBS, the sections were stained with 1 μg/ml 4′,6′-diamidino-2-phenylindole (DAPI, Invitrogen) in distilled water for 20 min [28]. After washing, the sections were mounted using Lab Vision™ PermaFluor™ (Invitrogen) medium under glass coverslips, then viewed and photographed in a Pannoramic DESK Digital Slide Scanner (3D HISTECH, Budapest, Hungary) [28]. The TUNEL mean fluorescence intensity was measured using Image-Pro Plus software (IPP, Maryland, USA).

### Data analysis

All statistical analyses were performed using GraphPad Prism 6.0 software (GraphPad Software Inc., San Diego, CA, USA). Mean ± standard error (SEM) values were calculated for three separate experiments, and two-tailed Student’s *t* tests were used to identify significant differences between groups. A value of *P* < 0.05 was considered to be statistically significant.

## Results

### Sequence analysis of *RhBcl-2* and *RhBax*

**Fig. 1:**
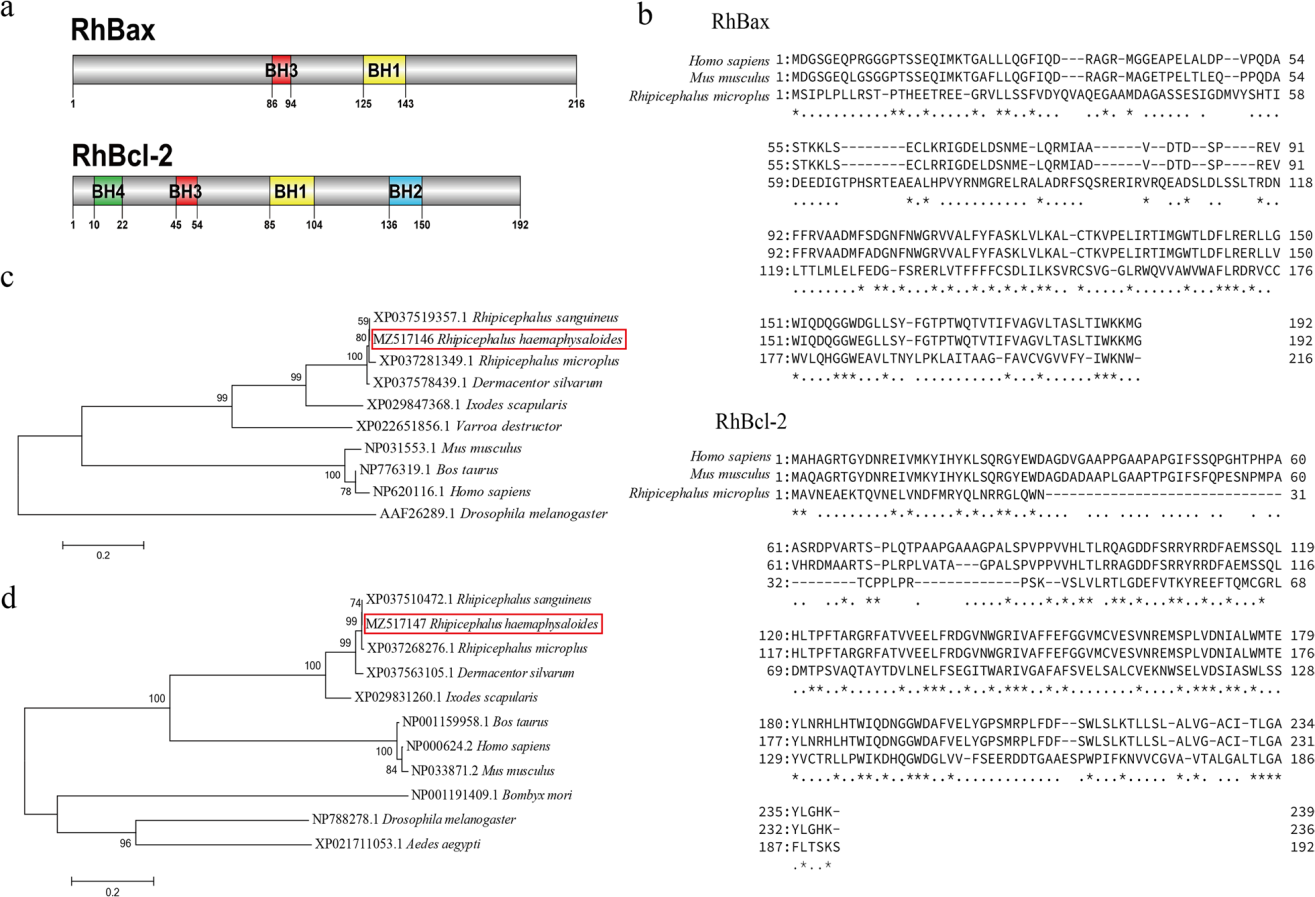
Sequence and structure analysis of *Rhipicephalus haemaphysaloides RhBcl-2* and *RhBax*. **a** Domain structure of *RhBcl-2* and *RhBax*. **b** Alignment of the deduced amino acid sequences of *RhBcl-2* (MZ517147) and *RhBax* (MZ517147); *Homo sapiens* Bcl-2: NP_000648.2; *Mus musculus* Bcl-2: NP_803129.2; *Homo sapiens* Bax: NP_001278357.1; *Mus musculus* Bax: NP_031553.1; **c** Phylogenetic tree of *RhBax* with the Bax of other species. **d** Phylogenetic tree of *RhBcl-2* with the Bcl-2 of other species. Bootstrap values after 500 simulations are shown at the branches. *RhBcl-2* and *RhBax* are marked with black triangle

We used nucleic acid sequences obtained from RNAseq. Specific cloning primers were designed according to the predicted sequences of *R. haemaphysaloides Bcl-2* and *Bax* (Additional file 1: Table S2). The ORF regions of Bcl-2 and Bax genes were cloned from the cDNA of fully engorged *R. haemaphysaloides* female salivary glands and named *RhBcl-2* and *RhBax*. *RhBcl-2* ORF has 576 bp and encodes a protein of 192 amino acid residues with a deduced molecular weight (MW) and theoretical isoelectric point (PI) of 21.4 kDa and 4.87, respectively. *RhBax* ORF has 648 bp and encodes a protein with 216 amino acid residues. It has a deduced MW of 24.6 kDa and a PI of 5.28. *RhBcl-2* had the conserved BH1, BH2, BH3, and BH4 domains, but *RhBax* had the conserved BH1 and BH3 domains (Fig. 1a).

The protein alignment and phylogenetic analyses showed the relationship between *RhBcl-2* or *RhBax* and Bcl-2 or Bax of other species (Fig. 1b–d). *RhBcl-2* and *RhBax* are highly homologous to their homologs in other ticks. Moreover, their similarity with mammals (*Homo sapiens*, *Mus musculus* and *Bos taurus*) are higher than with insects (*Drosophila melanogaster*, *Bombyx mori* and *Aedes aegypti*).

### Transcription and translation of *RhBcl-2* and *RhBax* profiles in different tissues, feeding periods and development stages

**Fig. 2:**
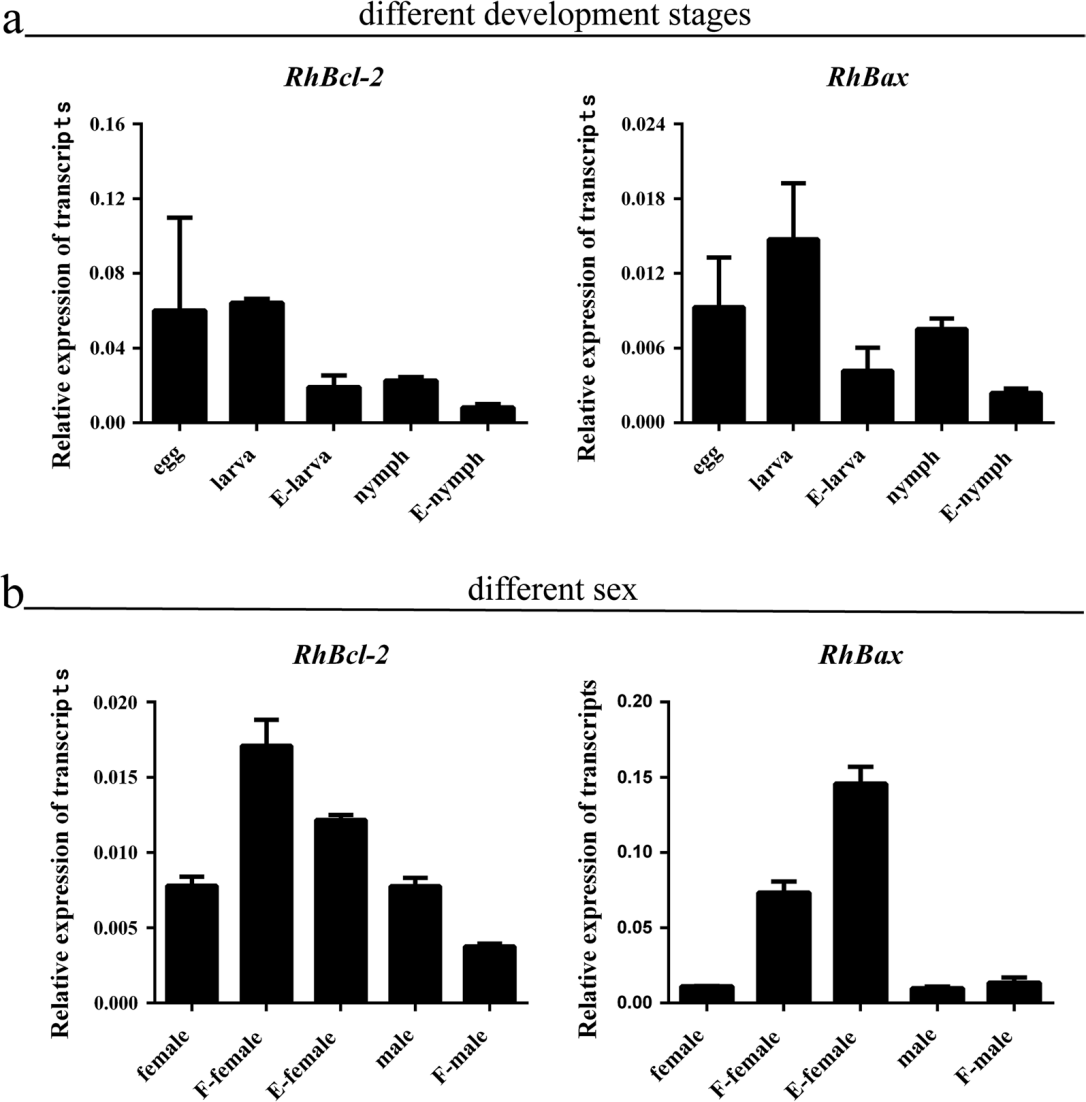
Transcription analysis of *RhBcl-2* and *RhBax* in *Rhipicephalus haemaphysaloides*, according to developmental stage and sex. **a** RT-qPCR analysis of *RhBcl-2* and *RhBax* gene expression during different developmental stages. **b** RT-qPCR analysis of *RhBcl-2* and *RhBax* gene expression according to sex. (F: fed, E: engorged)

The cDNA of eggs, larvae (unfed and engorged), and nymphs (unfed and engorged) were subjected to RT-qPCR to evaluate the expression profiles of *RhBcl-2* and *RhBax* genes (Fig. 2a). The cDNA of unfed adults (females and males), fed adults (females and males), and engorged female ticks were used to determine the sex-specific profile of *RhBcl-2* and *RhBax* genes (Fig. 2b). The transcription level of *RhBax* increased during feeding in females.

**Fig. 3:**
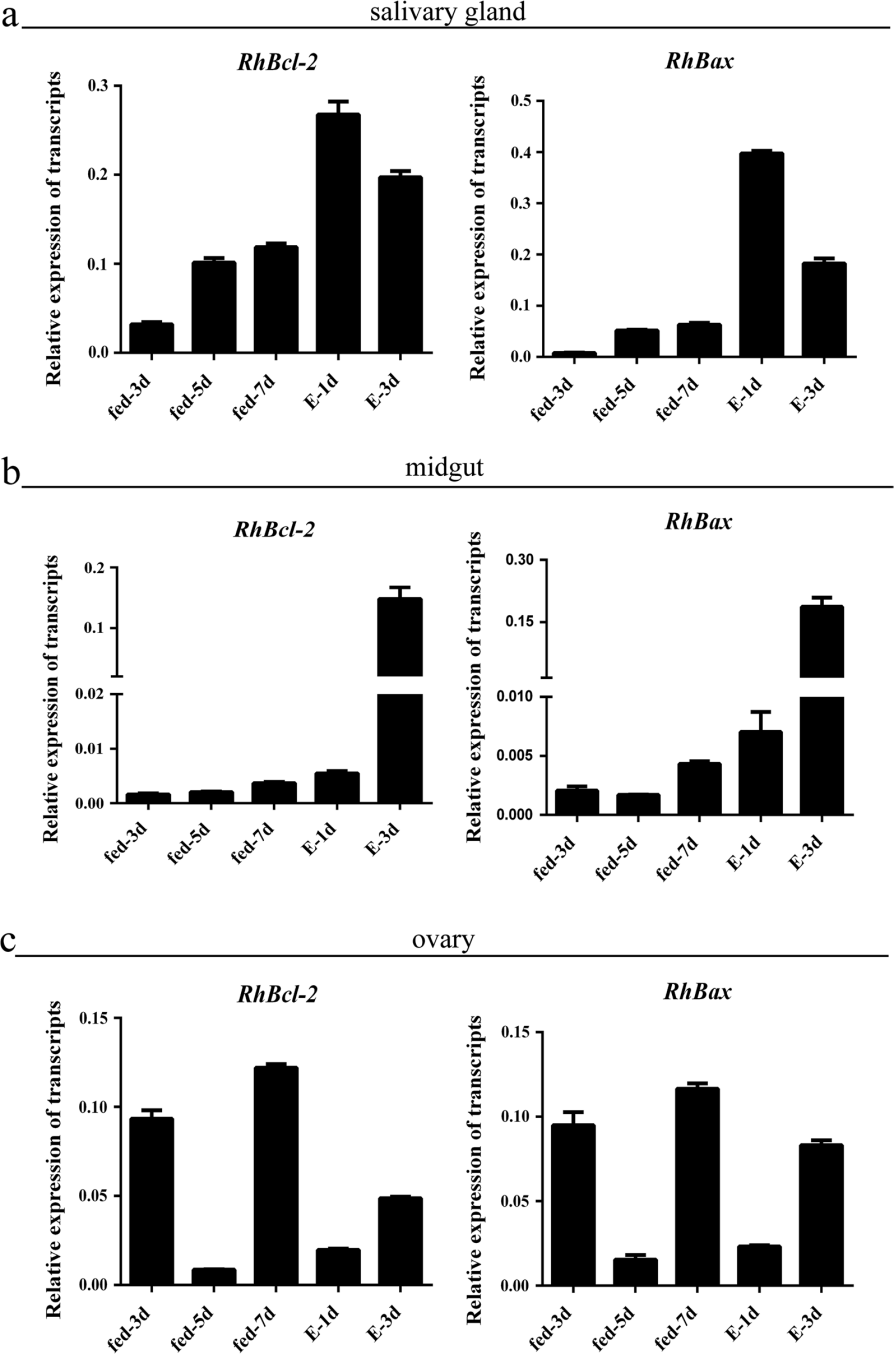
Transcription analysis of *RhBcl-2* and *RhBax* in *Rhipicephalus haemaphysaloides* in different tissues. **a**–**c** Transcription of *RhBcl-2* and *RhBax* in different tissues of *R. haemaphysaloides* during feeding time. (E: engorged)

After microdissection, the cDNAs of salivary glands, ovaries, and midguts of adult females at different feeding times were analyzed for *RhBcl-2* and *RhBax* gene expression profiles (Fig. 3a–c). *RhBcl-2* and *RhBax* genes were expressed in the examined tick tissues during feeding. However, *RhBcl-2* and *RhBax* had the same expression trends in different organs.

**Fig. 4:**
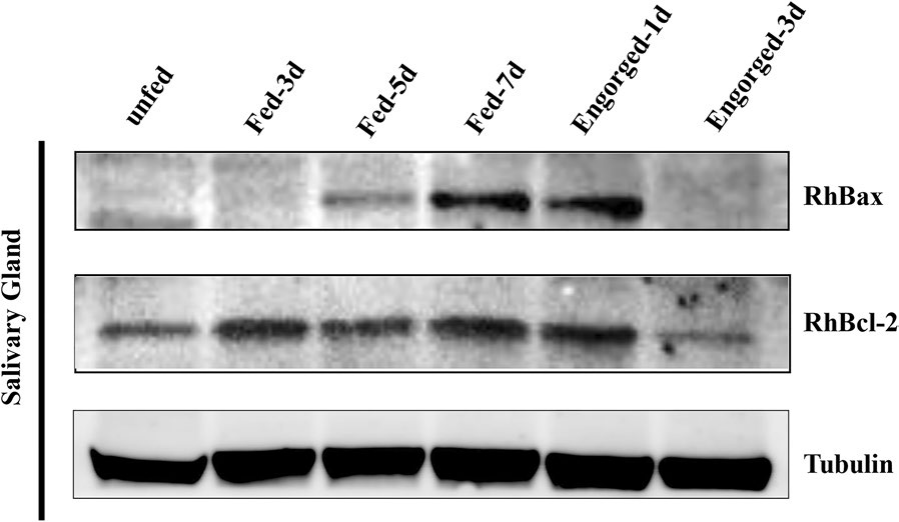
Western blot detection of RhBcl-2 and RhBax in salivary glands from female *Rhipicephalus haemaphysaloides* at different feeding times. Tick salivary glands were screened with sera anti-RhBcl-2 and RhBax, and anti-tubulin serum was used as the control

Western blot analysis was performed using RhBcl-2 and RhBax anti-sera (Fig. 4). RhBax was detected during the fast-feeding period to post-engorgement, and RhBcl-2 was in the feeding stages.

### Expression of RhBcl-2 and RhBax

The coding sequences of *RhBcl-2* and *RhBax* were cloned into prokaryotic expression vectors (pET-28a and pGEX-4T-1) to produce recombinant RhBcl-2 and RhBax. All of the recombinant proteins were expressed as inclusion bodies in *E. coli*.

**Fig. 5:**
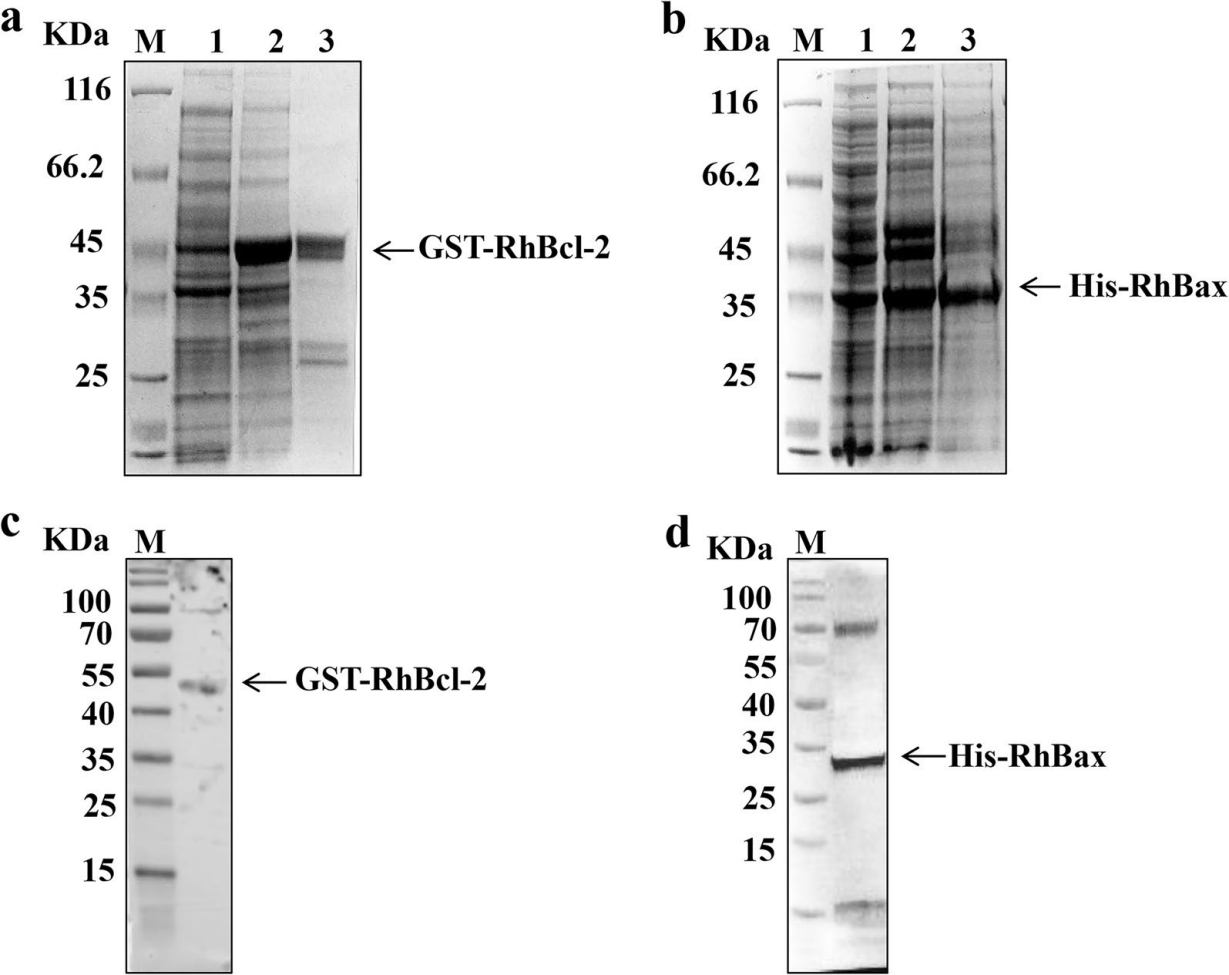
Expression of recombinant RhBcl-2 and RhBax. **a**, **b** Purification of recombinant RhBcl-2 and RhBax. Lane 1: M: the protein marker; Lane 2: non-induced; Lane 3: 1 mM IPTG induced expression for 8 h; Lane 4: recombinant protein after purification. **c**, **d** Western-blotting detection of RhBcl-2 and RhBax. Purified RhBcl-2 and RhBax were immunoblotted with the anti-sera against RhBcl-2 and RhBax, respectively

After solubilization and purification, His-RhBax (molecular weight: ~ 38 kDa) and GST-RhBcl-2 (molecular weight: ~ 50 kDa) were obtained, respectively (Fig. 5a, b). Specific polyclonal antibodies (PcAb) were created in response to the recombinant protein. Western blot analysis revealed that the two sera were able to identify the full recombinant proteins (Fig. 5c, d), and the fragment sizes were consistent with those observed by SDS-PAGE.

### RhBcl-2 binds with RhBax by BH3 domain

**Fig. 6:**
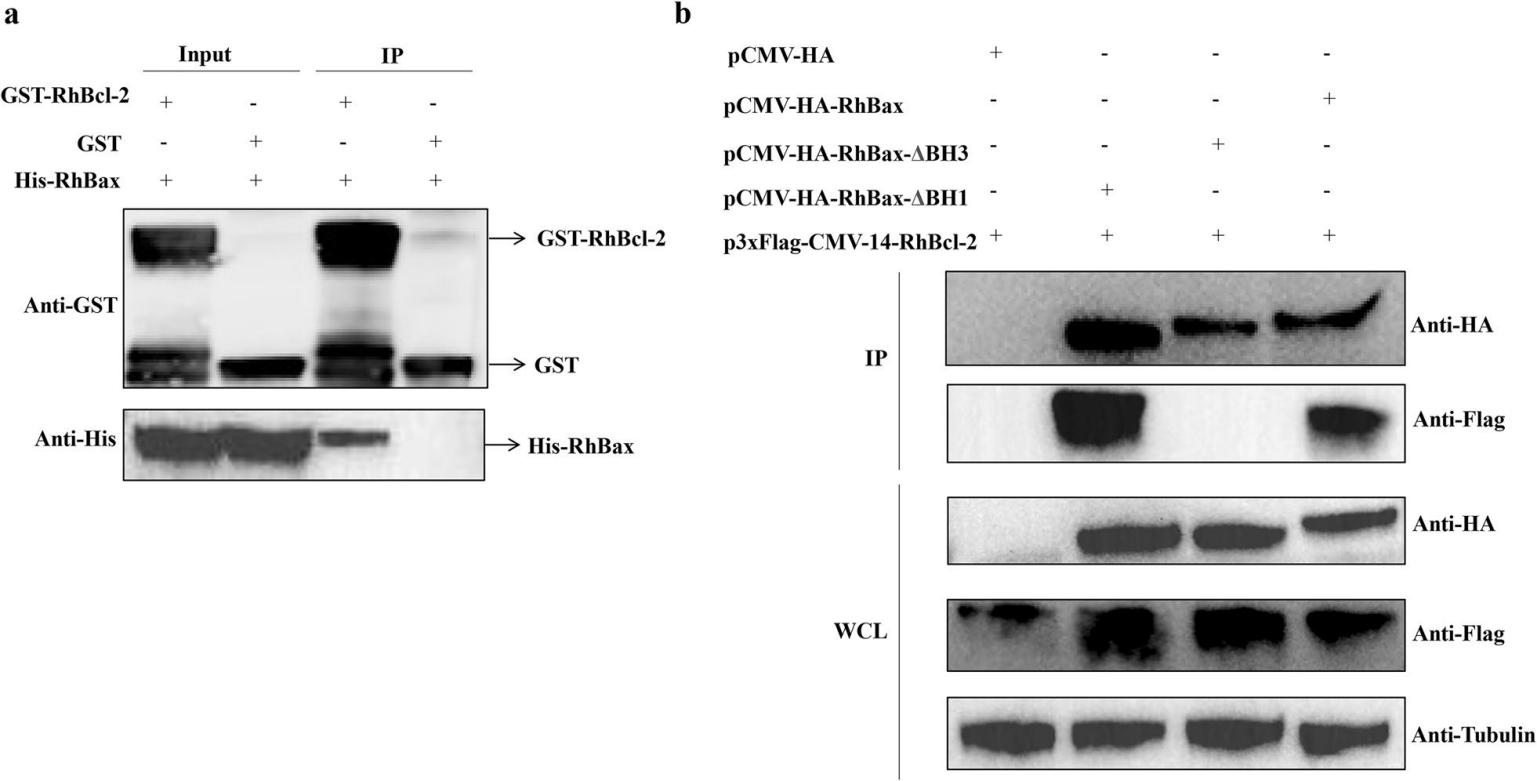
GST pull-down binding and co-transfection assays of the RhBcl-2 and RhBax. **a** The input protein (IP) samples showed pull-down binding assays of His-RhBax as prey with RhBcl-2 as a bait. The IP shows pull-down binding assays of His-RhBax with the GST-RhBcl-2. GST is used as a negative control. **b** Western blotting analysis of the interaction between RhBcl-2 and RhBax by co-transfection into HEK-293T cells. Lane 1: Cells co-transfected p3×Flag-CMV-14-RhBcl-2 with pCMV-HA vector. Lanes 2–4: Cells co-transfected p3×Flag-CMV-14-RhBcl-2 with pCMV-HA-RhBax-ΔBH1, pCMV-HARhBax-ΔBH3 and pCMV-HA-RhBax

To verify that RhBcl-2 interacts with RhBax, an in-vitro GST pull-down assay was used to confirm the interaction between His-RhBax and GST-RhBcl-2 (Fig. 6a). For this experiment, two recombinant proteins with either a GST-tag or His-tag, respectively (His-RhBax and GST-RhBcl-2), were produced from *E. coli* BL21. Immunoblotting showed that the GST and GST-RhBcl-2 were successfully pulled down with GST beads and that the GST-RhBcl-2 interacted directly with His-RhBax. GST as a negative control confirmed that the interaction of GST-RhBcl-2 and His-RhBax was specific.

Co-transfection of HEK 293T cells with plasmids containing *RhBcl-2* and *RhBax* genes resulted in co-expression of the proteins. The results also showed that RhBcl-2 and RhBax could interact with each other, but failed to combine without the BH3 domain (Fig. 6b).

### RNAi assay of *RhBcl-2* and RhBax

**Fig. 7:**
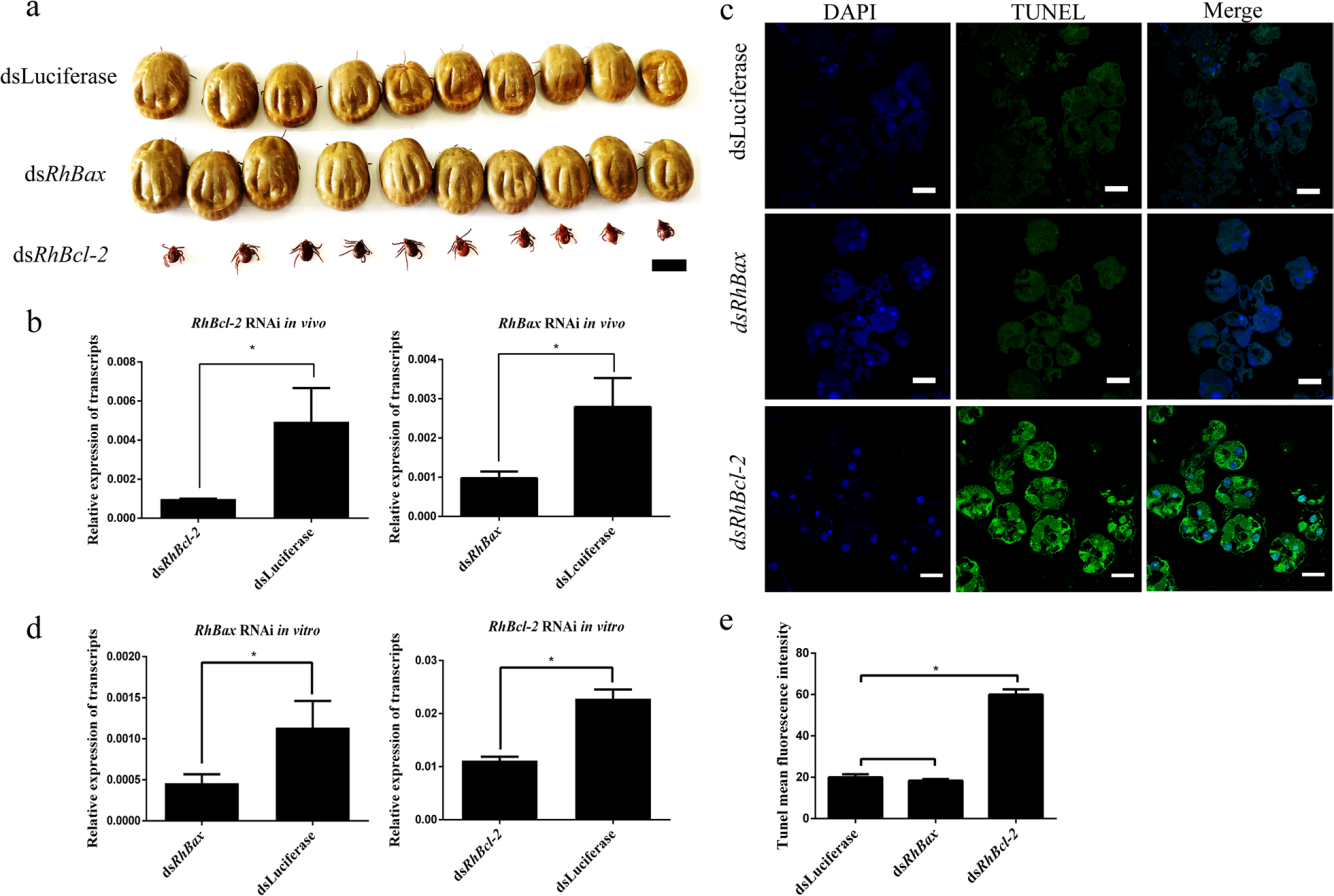
Biological effects of RNAi of *Rhipicephalus haemaphysaloides RhBcl-2* and *RhBax*. **a** Images comparing females inoculated with *dsRhBcl-2* and *RhBax* with control groups on day 7 of feeding. Ds*RhBcl-2* inhibited the blood-feeding process and resulted in females smaller than the controls. Scale bars: 5 mm. **b** Confirmation of *RhBcl-2* and *RhBax* silencing using RT-qPCR. Total RNA was extracted on feeding day 5 from whole bodies of female ticks injected with dsRNA. Bars represent mean relative expression of *RhBcl-2* and *RhBax*; error bars represent standard error. *P < 0.05, based on two-tailed Student’s *t* tests. **c** TUNEL staining assays of apoptosis levels after interference with the salivary glands by dsRNA of *RhBcl-2* and *RhBax*. DNA was stained with DAPI. Scale bars: 50 μm. **d** Confirmation of *RhBcl-2* and *RhBax* silencing using RT-qPCR. **e** Tunel mean fluorescence intensity showed the apoptosis levels. *P < 0.05

To identify the function of *RhBcl-2* and *RhBax*, the genes of *RhBcl-2* and *RhBax* were in vivo targets of RNA interference. The RT-qPCR analysis of ticks showed decreased levels of *RhBcl-2* and *RhBax* in *RhBcl-2*-dsRNA and *RhBax-*dsRNA injected groups compared to luciferase-dsRNA injected controls (Fig. 7b: *RhRhBcl-2* RNAi: *t*_(3)_ = 3.967, *P* = 0.0166; *RhBax* RNAi: *t*_(3)_ = 4.153, *P* = 0.0142; Fig. 7d: *RhRhBcl-2* RNAi: *t*_(3)_ = 10.80, *P* = 0.0004; *RhBax* RNAi: *t*_(3)_ = 3.433, *P* = 0.0265). There was no significant difference in the attachment rate between the experimental and control groups (Table 1), but females of group *RhBcl-2* did not reach the full engorgement stage (Fig. 7a) and died on the seventh day to the ninth days. Thus, the engorgement rate and death rates were significantly different compare with control group (Table 1). However, ticks injected with *RhBax*-dsRNA did not differ from the control groups at any feeding time (Table 1).

**Table 1:** Effect of knocking-down *RhBcl-2* and *RhBax* on tick feeding

Parameters	dsLuciferase	ds*RhBcl-2*		ds*RhBax*	
	% (*n*)	% (*n*)	Chi-square test	% (*n*)	Chi-square test
Attachment rate at 24 h	76.7 (60)	85.0 (60)	*χ*^*2*^ = 1.345, *df* = 1, *P* = 0.2462	70.0 (60)	*χ*^2^ = 0.6818, *df* = 1, *P* = 0.4090
Engorgement rate	73.3 (60)	0.0 (60)	*χ*^2^ = 69.74, *df* = 1, *P* < 0.0001	61.7 (60)	*χ*^2^ = 1.8610, *df* = 1, *P* = 0.1725
Death rate	20.0 (60)	95.0 (60)	*χ*^2^ = 69.50, *df* = 1, *P* < 0.0001	33.3 (60)	*χ*^2^ = 2.7270, *df* = 1, *P* = 0.0986
Egg laying rate	100.0 (30)	ND	NA	96.7 (30)	*χ*^2^ = 1.0170, *df* = 1, *P* = 0.3132
Hatchability rate	100.0 (30)	ND	NA	96.7 (30)	*χ*^2^ = 1.0170, *df* = 1, *P* = 0.3132

ND, no data; NA, not applicable; *n*, total number of specimens in each experiment

TUNEL assays showed that salivary gland interference by *RhBcl-2* dsRNA increased apoptosis levels, but *RhBax* was similar to the control (Fig. 7c, e).

## Discussion

*Rhipicephalus haemaphysaloides* is a three-host hard tick widely distributed in China. It is a carrier of several human pathogens [37–39]. The salivary glands of female ticks undergo degeneration after engorgement [3]. Apoptosis plays a role in the process while caspase genes and apoptosis-related genes are upregulated [40]. Our study identified and characterized two Bcl-2 family proteins (Bcl-2 and Bax) in *R. haemaphysaloides* and found them to be involved in salivary gland degeneration.

The Bcl-2 family proteins are also found in insects such as *Drosophila*, *Bombyx mori*, and *Anopheline mosquitoes*, and are involved in growth and metamorphosis [24, 41–43]. Ayllon [25] found that Bcl-2 may play a key role in the intrinsic apoptosis of *Ixodes scapularis*, but other Bcl-2 family proteins and the apoptosis mechanism are unknown. We found that *RhBcl-2* and *RhBax* are involved in salivary gland degeneration in *R. haemaphysaloides*. Sequence analysis demonstrated that *RhBcl-2* and *RhBax* have conserved sequences characteristic of BH domains. The BH3 domain is a key domain for Bax, Bak, and Bok to promote apoptosis and combine anti-apoptosis proteins with proapoptotic proteins [44, 45]. A GST pull-down assay confirmed that RhBcl-2 and RhBax combined with each other. Co-transfection assays also showed that RhBcl-2 and RhBax could interact, but did not combine without the BH3 domain.

Salivary gland degeneration is triggered after the completion of female tick feeding [46]. According to the RT-qPCR results, *RhBcl-2* and *RhBax* had a high level of transcription in salivary glands during tick engorgement, as determined by western blot. These findings suggest that the roles of RhBcl-2 and RhBax are related to salivary glands apoptosis and degeneration.

RNA interference was used to investigate the physiological roles of *RhBcl-2* and *RhBax*. TUNEL staining was used to evaluate the rate of DNA fragmentation in degenerated tick salivary glands. By interference with *RhBcl-2*, the TUNEL-positive staining rate increased significantly when compared to the control group. These results demonstrated that *RhBcl-2* functions in preventing apoptosis during salivary gland degeneration. Bax is a proapoptotic protein in the Bcl-2 protein family [47]. However, interference with *RhBax* had no significant effect when compared to the control group.

*RhBcl-2* silencing produced a most significant phenotype alteration in ticks during blood feeding compared to *RhBax* and the control groups. After injection with *dsRhBcl-2*, the female ticks were unable to feed and engorge until they died. These data illustrated the importance of *RhBcl-2* during blood feeding. Consequently, *RhBcl-2* functioned to protect cell survival, promote normal organ functioning, and ensure that ticks take in sufficient amount of blood in this stage. The function of the *RhBax* protein remains unclear.

## Conclusions

In conclusion, two Bcl-2 family molecules were identified in ticks, and the roles of *RhBcl-2* and *RhBax* in tick physiology were investigated. These findings enabled advanced studies of tick apoptosis. *RhBcl-2* and *RhBax*, like Bcl-2 and Bax in mammals, work together to regulate apoptosis in tick salivary glands. The knockdown of *RhBcl-2* in vivo inhibited blood feeding, indicating that RhBcl-2 has the potential to be used as a tick vaccine protein.

## Supplementary Information

**Additional file 1: Table S1**. Primers used for quantitative real-time polymerase chain reactions of *Rhipicephalus haemaphysaloides RhBcl-2* and *RhBax* genes. **Table S2**. Primers for *Rhipicephalus haemaphysaloides* RhBcl-2 and RhBax ORF cloning. **Table S3**. Primers used for overlap extension (SOE) of recombinant PCR to delete BH domain. **Table S4**. Primers for RNAi of *Rhipicephalus haemaphysaloides RhBcl-2* and *RhBax* genes.

## Data Availability

All data generated or analysed during this study are included in this published article and its Additional file 1: Tables S1–S4.

## Abbreviations

TBPs: Tick-borne pathogens
PCD: Programmed cell death
PcAb: Polyclonal antibodies
RT-qPCR: Reverse transcription quantitative polymerase chain reaction
PBS: Phosphate-buffered saline
TBS: Tris-buffered saline
SDS-PAGE: Sodium dodecyl sulfate polyacrylamide gel electrophoresis
IPTG: Isopropyl β-d-1-thiogalactopyranoside
DAPI: 4′,6′-Diamidino-2-phenylindole
TUNEL: Terminal deoxynucleotidyl transferase dUTP nick-end labeling
BH: Bcl-2 homology
