# Supplementary material for: Identification of the Bcl-2 and Bax homologs from Rhipicephalus haemaphysaloides and their function in the degeneration of tick salivary glands
Source: Parasit Vectors. 2021 Aug 4;14:386. doi: 10.1186/s13071-021-04879-z (PMC8336254; doi:10.1186/s13071-021-04879-z)
Supplement: Supplementary file 1 — Additional file 1: Table S1. Primers used for quantitative real-time polymerase chain reactions of Rhipicephalus haemaphysaloides RhBcl-2 and RhBax genes. Table S2. Primers for Rhipicephalus haemaphysaloides RhBcl-2 and RhBax ORF cloning. Table S3. Primers used for overlap extension (SOE) of recombinant PCR to delete BH domain. Table S4. Primers for RNAi of Rhipicephalus haemaphysaloides RhBcl-2 and RhBax genes. [file 13071_2021_4879_MOESM1_ESM.docx]

**Additional file 1: Table S1.** Primers used for quantitative real-time polymerase chain reactions of *Rhipicephalus haemaphysaloides* *RhBcl-2* and *RhBax* genes.

| Name | Sequence |
| --- | --- |
| ELF1A-F | CGTCTACAAGATTGGTGGCATT |
| ELF1A-R | CTCAGTGGTCAGGT TGGCAG |
| *RhBcl-2*-F | TGGGCCACGGAGGGAGTCATGTCGA |
| *RhBcl-2*-R | TCGTGTAGCCGGTGAGCGGTTGAG |
| *RhBax*-F | TTCTCCGCTCTTTCCTGTATTT |
| *RhBax*-R | GAACTTGTGCAGCTTCAATGAG |

*Abbreviations*: F, forward primer; R, reverse primer.

**Additional file 1: Table S2.** Primers for *Rhipicephalus haemaphysaloides* *RhBcl-2* and *RhBax* ORF cloning.

| Name | Sequence |
| --- | --- |
| PGEX-4T-1-RhBcl-2-F | CTGGTTCCGCGTGGATCCATGGCCGTCAACGAGGCCG |
| PGEX-4T-1-RhBcl-2-R | GAGTCGACCCGGGAATTCCGACTTGGACGTGAGGAAGGC |
| pET-30a-Rhbax-F | GGCTGATATCGGATCCATGAGCATTCCCCTCCCACTACT |
| pET-30a-Rhbax-R | GTGGTGGTGCTCGAGCCAGTTCTTCCATATGTAAAATACAACACCC |
| pCMV-HA-RhBax-F | CCATGGAGGCCCGAATTCGGATGAGCATTCCCCTCCCACTAC |
| pCMV-HA-RhBax-F | CGCGGCCGCGGTACCTCGAGACCAGTTCTTCCATATGTAAAATACAACACC |
| p3xFlag-cMV-RhBcl-2-F | AGCTTGCGGCCGCGAATTCAATGGCCGTCAACGAGGCCGAGAAG |
| p3xFlag-cMV-RhBcl-2-R | TTTGTAGTCAGCCCGGGATCCCGACTTGGACGTGAGGAAGGCGC |

*Abbreviations*: F, forward primer; R, reverse primer.

**Additional file 1: Table S3.** Primers used for overlap extension (SOE) of recombinant PCR to delete BH domain.

| Name | Sequence |
| --- | --- |
| pCMV-HA-RhBax-△BH1-F1 | CCATGGAGGCCCGAATTCGGATGAGCATTCCCCTCCCACTAC |
| pCMV-HA-RhBax-△BH1-R1 | GACTGACTTCAGGATGAGGTCGCTGAGCATGAGCGTT |
| pCMV-HA-RhBax-△BH1-F2 | AGACAACCTCACAACGCTCATGCTCAGCGACCTCATCCT |
| pCMV-HA-RhBax-△BH1-R2 | CGCGGCCGCGGTACCTCGAGACCAGTTCTTCCATATGTAAAATACAACACC |
| pCMV-HA-RhBax-△BH3-F1 | CCATGGAGGCCCGAATTCGGATGAGCATTCCCCTCCCACTAC |
| pCMV-HA-RhBax-△BH3-R1 | AGTGAACTCCTCGCGGTATTTGGTTACAAGAGACACTT |
| pCMV-HA-RhBax-△BH3-F2 | GACCTTCCAAAGTGTCTCTTGTAACCAAATACCGCGA |
| pCMV-HA-RhBax-△BH3-R2 | CGCGGCCGCGGTACCTCGAGACCAGTTCTTCCATATGTAAAATACAACACC |

*Abbreviations*: F, forward primer; R, reverse primer.

**Additional file 1: Table S4.** Primers for RNAi of *Rhipicephalus haemaphysaloides* *RhBcl-2* and *RhBax* genes.

| Name | Sequence |
| --- | --- |
| *RhBcl-2* dsRNA-S1 | GGATCCTAATACGACTCACTATAGGACTGCGGACGCTGGGGGATG |
| *RhBcl-2* dsRNA-A1 | GGTCTTGGAGCACCGAGCAGAGGGC |
| *RhBcl-2* dsRNA-S2 | GGAGCATTTTTGGGGGAATCTTTGG |
| *RhBcl-2* dsRNA-A2 | GGATCCTAATACGACTCACTATAGGCCGAGCGTGAGGGCACCGAG |
| *RhBax* dsRNA-S1 | GGATCCTAATACGACTCACTATAGGACACCGACGCACGAGGAAACAC |
| *RhBax* dsRNA-A1 | GGGATACCGTGGCACTGTTT |
| *RhBax* dsRNA-S2 | AGTTTGGAGCCTCGGTTGAC |
| *RhBax* dsRNA-A2 | GGATCCTAATACGACTCACTATAGGAACGCAGTCCGCCCACAGAG |

*Abbreviations*: S, forward primer; A, reverse primer, the sequence in underlined indicates the sequence of the T7 promoter.
